# Asymptomatic pancreatic enlargement without pancreatic enzyme elevation: a rare case of immune checkpoint inhibitor-associated pancreatitis

**DOI:** 10.1093/gastro/goae064

**Published:** 2024-06-10

**Authors:** Yasuki Hori, Tatsuya Kawai, Aya Naiki-Ito, Itaru Naitoh, Michihiro Yoshida, Akihisa Kato, Hiromi Kataoka

**Affiliations:** Department of Gastroenterology and Metabolism, Nagoya City University Graduate School of Medical Sciences, Nagoya, Japan; Department of Radiology, Nagoya City University Midori Municipal Hospital, Nagoya, Japan; Department of Experimental Pathology and Tumor Biology, Nagoya City University Graduate School of Medical Sciences, Nagoya, Japan; Department of Gastroenterology, Nagoya City University Midori Municipal Hospital, Nagoya, Japan; Department of Gastroenterology and Metabolism, Nagoya City University Graduate School of Medical Sciences, Nagoya, Japan; Department of Gastroenterology and Metabolism, Nagoya City University Graduate School of Medical Sciences, Nagoya, Japan; Department of Gastroenterology and Metabolism, Nagoya City University Graduate School of Medical Sciences, Nagoya, Japan

## Introduction

Immune checkpoint inhibitors (ICIs) have shown clinical efficacy in the treatment of various malignancies [1, 2]. The grading system for ICI-associated pancreatitis is based on elevated levels of pancreatic enzymes, radiological findings, and/or clinical findings [3]. Most cases of ICI-associated pancreatitis exhibit symptoms and/or pancreatic enzyme elevation, but imaging changes are often not dramatic [4]. Here, we presented the case of a patient with asymptomatic pancreatic enlargement without the enzyme elevation. She was diagnosed with ICI-associated pancreatitis via endoscopic ultrasound-guided fine-needle biopsy (EUS-FNB) and treated with immunosuppressive therapy.

## Case report

**Figure 1.**
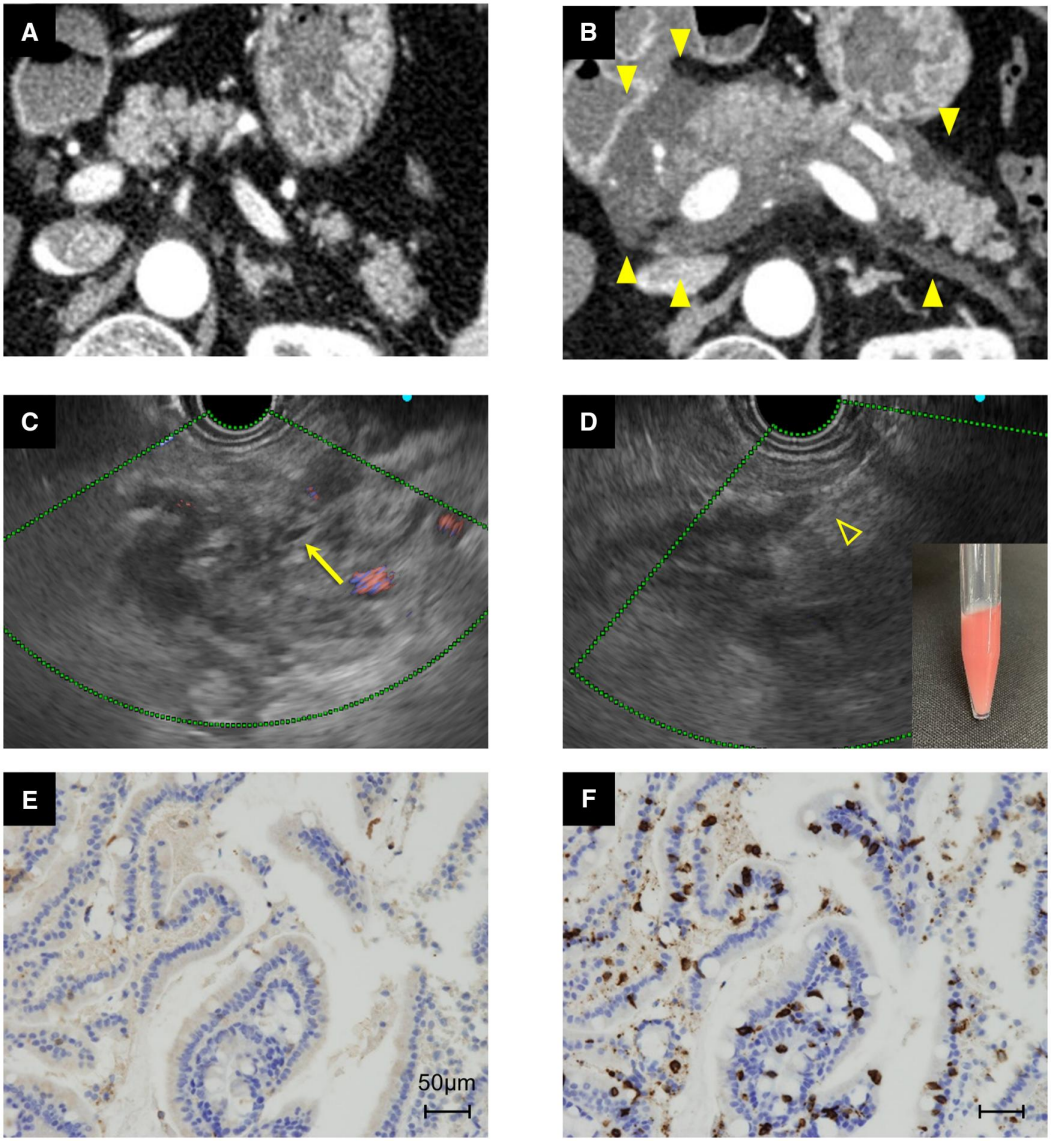
Computed tomography and endoscopic ultrasound images and pathological findings of ICI-associated pancreatitis. (A) Contrast-enhanced computed tomography images before ICI therapy. (B) Diffuse pancreatic enlargement developed after 20 cycles of nivolumab. These images show parenchymal enlargement with reduced contrast enhancement and/or perifocal fatty oedema. (C) Endoscopic ultrasound images and findings of ICI-associated pancreatitis. The head of the pancreas was swollen, with scattered hyperechoic and hypoechoic areas, but the main pancreatic duct was not dilated (yellow arrow). (D) The head of the pancreas was biopsied using a 22-gauge needle and a slightly reddish milky-white fluid was obtained by aspiration. The fluid solidified at room temperature. (E) and (F) Pathological findings of the head of the pancreas. Immunohistochemical findings of CD4+ (E) and CD8+ (F) are presented. The CD8+/CD4+ ratio was high, consistent with the findings of immune-related adverse events. CD = cluster of differentiation, ICI = immune checkpoint inhibitor.

A 69-year-old woman was diagnosed with angiosarcoma and treated with nivolumab, an inhibitor of programmed cell death receptor-1. She had a history of systemic sclerosis and did not consume alcohol or smoke. The imaging findings of the pancreas were normal before ICI treatment (Figure 1A). Two months later, the pancreas head was enlarged, but there was no abdominal pain or pancreatic enzyme elevation (lipase 44 U/L, C-reactive protein [CRP] 0.14 mg/dL). After 15 months, the swelling increased and extended throughout the pancreas (Figure 1B). The patient remained asymptomatic and did not exhibit pancreatic enzyme elevation (lipase 40 U/L, CRP 0.13 mg/dL). The levels of serum immunoglobulin G4 (26.6 mg/dL) and CA19-9 (35.3 U/mL) were normal. She could not undergo magnetic resonance imaging because of claustrophobia.

Figure 1C shows the EUS images of the pancreas. The head was swollen, with scattered hyperechoic and hypoechoic areas, but the main duct was not dilated (yellow arrow). The time-series variation in the imaging findings was further investigated by performing an EUS-FNB of the head of the pancreas (Figure 1D). Hematoxylin and eosin staining showed epithelial cells of the pancreatic duct clustered with inflammatory cells. CD4+ (Figure 1E) and CD8+ (Figure 1F) were also assessed by immunohistochemistry (scale bar = 50 mm). The CD8+/CD4+ ratio was high, consistent with an immune-related adverse event. Other causes of pancreatitis, such as alcohol intake, gallstones, hypertriglyceridemia, viral infection, genetic predisposition, tumors, and anatomical variants, were excluded in the patient. Patient data were abstracted from the electronic medical records including blood tests and image findings.

The patient’s clinical course was summarized (Supplementary Figure 1), including the pancreatic volume and the levels of serum lipase and CRP; notably, these levels remained normal throughout the clinical course, despite a progressive increase in pancreatic volume. The patient developed ICI-pneumonitis with dyspnoea, prompting the administration of immunosuppressive agents. Subsequently, the pancreatic swelling resolved. The findings also support the notion that the pancreatic swelling was related to ICI-associated pancreatitis. After treatment with immunosuppressive agents, pseudocysts appeared within the body of the pancreas, evidence of chronic changes in the pancreas. Despite administration of various immunosuppressive agents, the patient finally died of respiratory failure.

## Discussion

The National Comprehensive Cancer Network (NCCN) guidelines define the grade of ICI-associated pancreatitis based on pancreatic enzyme elevation, radiological findings, or clinical manifestations [3]. In a recent systematic review and meta-analysis [5], the incidence of asymptomatic lipase elevation after ICI therapy was 2.7% and that of grade 2 pancreatitis was 1.9%. In most cases of ICI-associated pancreatitis, patients have symptoms or pancreatic enzyme elevation. However, our patient did not have symptoms or pancreatic enzyme elevation throughout the clinical course. After immunosuppressive treatment, imaging changes suggestive of chronic disease in the pancreas appeared, but the patient had no symptoms related to chronic pancreatitis, such as steatorrhea or impaired glucose tolerance. Previous reports [6, 7] have also noted that the presence of chronic changes on radiological imaging is not always associated with endocrine or exocrine dysfunction. Further studies are needed to clarify the long-term changes in pancreatic function after ICI administration.

In most patients with ICI-associated pancreatitis, the radiological findings are similar to those of mild acute pancreatitis. Das *et al.* [8] assessed contrast-enhanced computed tomography images of 25 patients receiving ICI therapy and with evidence of pancreatitis. Diffuse pancreatic enlargement was seen in 56.0% of the patients and focal enlargement was observed in 44.0%. A pattern consistent with acute pancreatitis and with autoimmune pancreatitis was present in 20/25 (80%) and 4/25 (16%) patients, respectively. Our patient had diffuse pancreatic enlargement but it was not the same as typical acute or autoimmune pancreatitis. Based on the slightly reddish milky-white fluid obtained by EUS-guided fine-needle aspiration (Figure 1D), inflammation coupled with pancreatic damage, similar to the pathophysiological findings of acute pancreatitis, was likely.

EUS may play an important diagnostic role [9], but reports of EUS imaging findings of ICI-associated pancreatitis are rare. In our patient, the findings included swelling of the pancreatic head (Figure 1C) with scattered hyperechoic and hypoechoic areas but without dilation of the main pancreatic duct. Definitive ultrasound imaging features of ICI-associated pancreatitis have not been established. EUS is somewhat useful for excluding space-occupying lesions, but EUS-FNB allows the acquisition of tissue for histopathological diagnosis. Although information on the histopathological findings of ICI-associated pancreatitis is limited, an elevated CD8+/CD4+ ratio may indicate features consistent with immune-related adverse events [10], similar to the findings in liver biopsies.

Currently, there is no optimal treatment for ICI-associated pancreatitis. NCCN guidelines [3] advise against intervention in patients with asymptomatic pancreatic enzyme elevation. The guidelines state that ICI therapy can be continued in asymptomatic patients, accompanied by close monitoring of pancreatic enzyme levels. Permanent discontinuation of ICI therapy is recommended for patients with severe ICI-associated pancreatitis. In moderate-to-severe cases, steroid therapy (prednisone/methylprednisolone at 0.5–1.0 mg/kg/day, tapered slowly over 4–6 weeks) is recommended. But some researchers [11] have argued that steroids have no value in the management of ICI-associated pancreatitis. They indicated that steroids cannot prevent either short-term complications (e.g. pancreatitis-specific symptoms, pseudocysts) or long-term complications (e.g. diabetes, chronic pancreatitis). Thus, the usefulness of steroids in the treatment of ICI-associated pancreatitis remains unclear, and other therapeutics might be developed in future. Guidelines for other immune-related adverse events [12], such as ICI-related hepatitis, suggest adding mycophenolate mofetil and/or tacrolimus for steroid refractory cases. Kramer *et al.* [13] used those drugs as maintenance therapy for steroid refractory ICI-associated pancreatitis with favorable outcomes. Clinicians should consider the fact that patients receiving ICI therapy have advanced malignancies, and future studies should evaluate the optimal treatment timing and strategy.

In conclusion, we described a rare case of ICI-associated pancreatitis, with asymptomatic pancreatic enlargement but without pancreatic enzyme elevation. Our findings could lead to more precise diagnoses and treatments of ICI-associated pancreatitis.

## Supplementary Data 

Supplementary data is available at *Gastroenterology Report* online.

## Authors’ Contributions

Y.H. and I.N. wrote the paper and prepared figures; Y.H., T.K., A.N., M.Y., and A.K. analyzed and interpreted the data; T.K., A.N., and H.K. revised the paper for important intellectual content. All authors read and approved the final manuscript.

## Funding 

This work was supported by JSPS KAKENHI Grant Number [22K16027] and The Hori Sciences and Arts Foundation Grant Number [JOSE205003]. The funder had no role in the study design, data collection, data analysis, data interpretation, or writing of the paper.

## Supplementary Material

goae064_Supplementary_Data
