# Supplementary figures and images for: Asymptomatic pancreatic enlargement without pancreatic enzyme elevation: a rare case of immune checkpoint inhibitor-associated pancreatitis
Source: Gastroenterol Rep (Oxf). 2024 Jun 10;12:goae064. doi: 10.1093/gastro/goae064 (PMC11165310; doi:10.1093/gastro/goae064)

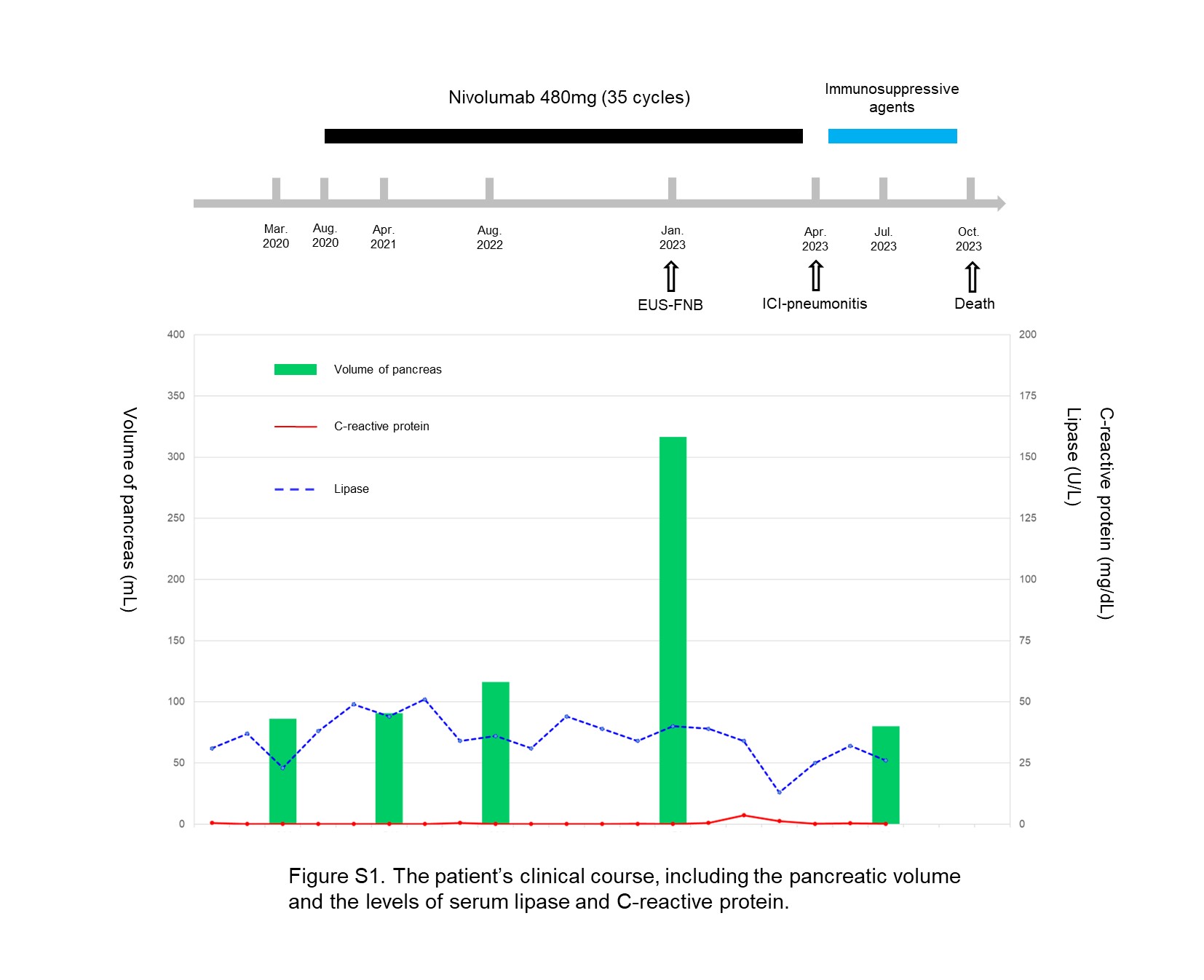

Supplement: goae064_Supplementary_Data [file goae064_supplementary_data.jpeg]
